# The effect of prone positioning on mortality in patients with acute respiratory distress syndrome: a meta-analysis of randomized controlled trials

**DOI:** 10.1186/cc13896

**Published:** 2014-05-28

**Authors:** Shu Ling Hu, Hong Li He, Chun Pan, Ai Ran Liu, Song Qiao Liu, Ling Liu, Ying Zi Huang, Feng Mei Guo, Yi Yang, Hai Bo Qiu

**Affiliations:** 1 Department of Critical Care Medicine, Zhongda Hospital, and School of Medicine, Southeast University, No.87, Dingjiaqiao Road, Nanjing, Gulou District 210009, China

## Abstract

**Introduction:**

Prone positioning (PP) has been reported to improve the survival of patients with severe acute respiratory distress syndrome (ARDS). However, it is uncertain whether the beneficial effects of PP are associated with positive end-expiratory pressure (PEEP) levels and long durations of PP. In this meta-analysis, we aimed to evaluate whether the effects of PP on mortality could be affected by PEEP level and PP duration and to identify which patients might benefit the most from PP.

**Methods:**

Publications describing randomized controlled trials (RCTs) in which investigators have compared prone and supine ventilation were retrieved by searching the following electronic databases: PubMed/MEDLINE, the Cochrane Library, the Web of Science and Elsevier Science (inception to May 2013). Two investigators independently selected RCTs and assessed their quality. The data extracted from the RCTs were combined in a cumulative meta-analysis and analyzed using methods recommended by the Cochrane Collaboration.

**Results:**

A total of nine RCTs with an aggregate of 2,242 patients were included. All of the studies received scores of up to three points using the methods recommended by Jadad *et al*. One trial did not conceal allocation. This meta-analysis revealed that, compared with supine positioning, PP decreased the 28- to 30-day mortality of ARDS patients with a ratio of partial pressure of arterial oxygen/fraction of inspired oxygen ≤100 mmHg (*n* = 508, risk ratio (RR) = 0.71, 95 confidence interval (CI) = 0.57 to 0.89; *P* = 0.003). PP was shown to reduce both 60-day mortality (*n* = 518, RR = 0.82, 95% CI = 0.68 to 0.99; *P* = 0.04) and 90-day mortality (*n* = 516, RR = 0.57, 95% CI = 0.43 to 0.75; *P* < 0.0001) in ARDS patients ventilated with PEEP ≥10 cmH_2_O. Moreover, PP reduced 28- to 30-day mortality when the PP duration was >12 h/day (*n* = 1,067, RR = 0.73, 95% CI = 0.54 to 0.99; *P* = 0.04).

**Conclusions:**

PP reduced mortality among patients with severe ARDS and patients receiving relatively high PEEP levels. Moreover, long-term PP improved the survival of ARDS patients.

## Introduction

Acute respiratory distress syndrome (ARDS) is a common, serious condition of critically ill patients and a major cause of death in ICUs. Although numerous approaches have been employed to improve the effects of ventilation [1-3], including protective ventilator setting strategies and paralytic agents, the reported mortality rate of ARDS patients continues to be as high as 40% [4]. The high resource consumption associated with this severe disease results in a heavy burden to society.

Prone positioning (PP) is a relatively simple method that has been shown to improve gas exchange and oxygenation in ARDS patients [5,6]. Several mechanisms [7-10] have been suggested to explain these effects: (1) improvement in regional ventilation, (2) redistribution of perfusion mainly related to the horizontal axis, (3) greater homogeneity of ventilation/perfusion ratios, (4) recruitment of perfused tissue from dorsal regions that exceeds ventral derecruitment and (5) increases in lung volume and alveolar recruitment due to unloading of diaphragmatic movement in the prone position. However, during the past few years, no significant changes in the mortality rate of ARDS patients have been observed, despite improvements in oxygenation [5,6].

More recently, researchers in several studies have shown that PP can improve the survival of patients with severe hypoxemic ARDS [11-13]. It has also been suggested that long durations of PP should be applied in ARDS patients with low lung recruitability [9,11,12]. In addition, ventilator settings, particularly PEEP levels, may have an impact on the effects of PP [14]. Therefore, in this meta-analysis, we aimed to evaluate whether the effects of PP on mortality could be affected by PEEP levels and by the PP duration, as well as which patients might benefit the most from PP.

## Methods

### Data sources and search strategies

Reports of randomized controlled trials (RCTs) of PP in ARDS patients were retrieved by searching the following data sources: PubMed/MEDLINE, the Cochrane Library, the Web of Science and Elsevier Science (inception to May 2013). The following keywords were used: (“prone position” OR “body posture” OR “body position” OR “prone positioning”) AND (“acute respiratory distress syndrome” OR “lung injury” OR “respiratory failure” OR “ALI” OR “ARDS”)*.* Adult and pediatric populations were included in this literature search, and we restricted the literature language to English.

### Study selection

Two investigators assessed the retrieved studies, and they included the titles, abstracts and citations independently for possible consideration. The reviewers evaluated the studies for inclusion based on the criteria presented below, and they resolved any differences by consensus. The investigators selected the retrieved studies that fulfilled the inclusion and exclusion criteria. Because this is a meta-analysis of previously published studies, no ethical approval or patient consent was required.

#### Inclusion criteria

Studies were included if the following criteria were present: (1) the study was a trial comparing only the prone position with the supine position in patients with acute respiratory failure, acute lung injury or ARDS; (2) the definition of ARDS or the diagnostic criteria for ARDS were similar; (3) the study was a clinical RCT; (4) 28- to 30-day mortality data were available or ICU mortality, 60-day mortality or 90-day mortality was presented; and (5) the numbers of patients in the prone and supine positions were provided.

#### Exclusion criteria

Studies were excluded from the meta-analysis according to the following exclusion criteria: (1) the article was an editorial, review, letter or other type of publication not based on original research; (2) the full text was unavailable; (3) the study did not include extractable outcomes or mortality data; (4) the study was not an RCT; (5) the trial did not use supine positioning (SP) as a control (for example, the lateral position was used as a control); and 6) the trial applied significantly different adjunct interventions to the prone position and supine position groups (for example, the supine position group received high-frequency oscillatory ventilation, but the prone position group did not).

### Quality assessment

Two of the researchers (SLH and HLH) independently evaluated the methodological quality of each trial using a 5-point scale described by Jadad *et al*. [15]. This instrument was used to assess the following three aspects: (1) the use of randomization, (2) the use of blinding and (3) the handling of withdrawals and dropouts. The same two researchers inspected the details of the randomization methods by assessing the quality of the allocation concealment (adequate, uncertain, inadequate or not used) according to the criteria of the Cochrane Collaboration [16].

### Data extraction

Our primary outcome was 28- to 30-day mortality. The secondary outcomes were ICU mortality, 60-day mortality and 90-day mortality. We abstracted the main information, including the numbers of patients in the prone and supine positions, the partial pressure of arterial oxygen/fraction of inspired oxygen (P/F) threshold ratio for patient enrollment, the application of PEEP, the PP duration and the nonsurviving populations in the prone and supine positions. Additional information was extracted, such as age, sex, ICU length of stay, days on mechanical ventilation (MV), number of consecutive days of PP, number of cases of organ dysfunction, plateau pressure and tidal volume (V_t_). The two researchers (SLH and HLH) independently extracted all of the data. Disagreements between the two investigators were resolved by discussion and consensus, and a third party was involved in this procedure when necessary.

### Data analysis and statistical methods

The κ statistic was used to assess agreement between the evaluators regarding trial selection and methodological quality assessment. The meta-analysis of the effects of PP on mortality in ARDS patients was conducted according to methods recommended for use with the Cochrane Collaboration’s RevMan software, version 5.2.3 (The Nordic Cochrane Center, Rigshospitalet, Copenhagen, Denmark). Statistical heterogeneity and inconsistency were measured and quantified using the Mantel–Haenszel (M-H) χ^2^ test and the *I*^2^ test of heterogeneity in RevMan. Obvious heterogeneity was predefined at *P* < 0.05 by Mantel–Haenszel χ^2^ test or *I*^2^ > 50%. In cases of significant heterogeneity (*P* < 0.05 or *I*^2^ > 50%), a random-effects model was used; otherwise, a fixed-effects model was applied. We report risk ratios (RRs) with 95% confidence intervals (CIs) for the dichotomous data and weighted mean differences with 95% CIs for the continuous data. Publication bias was evaluated by visual inspection of funnel plots. Because only nine studies were included in the meta-analysis, a linear regression test of funnel plot asymmetry (Egger’s test) [17] could not be performed.

## Results

### Search results

**Figure 1:**
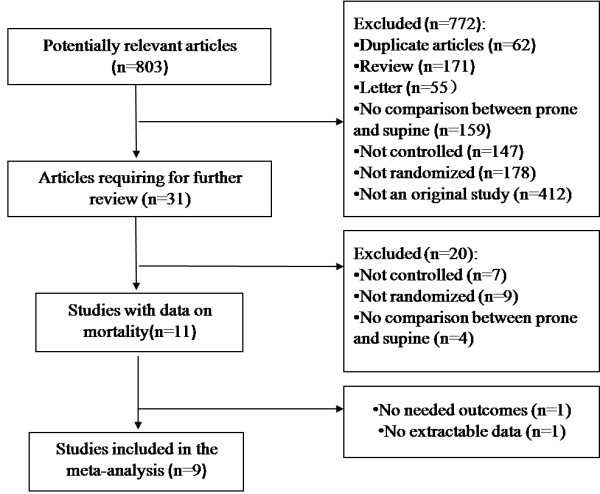
Flowchart of the meta-analysis.

We identified 803 potentially relevant articles from among 442 listed in PubMed/Medline, 28 in the Cochrane Library, 276 in the Web of Science and 57 in Elsevier Science. We retrieved 31 citations for detailed evaluation. Ultimately, nine prospective RCTs, including one pediatric study [18], fulfilled the inclusion criteria and were included in the cumulative meta-analysis. Figure 1 shows a flowchart of the studies that were assessed and excluded at different stages of the review.

### Trial characteristics and methodological quality

The included studies were published from 2001 to 2013 and enrolled an aggregate of 2,242 patients, including 1,150 patients in the prone position and 1,092 in the supine position. Table 1 and Additional file 1: Table S1 present the characteristics of all of the included patients. Basic information about the P/F thresholds for enrollment, PEEP levels, PP durations and V_t_ levels in each included trial was examined. Moreover, we recorded data on the primary outcomes (28- to 30-day mortality) of patients with P/F ≤ 300 mmHg and the subgroups of patients with P/F ≤ 100 mmHg and 100 mmHg < P/F ≤ 200 mmHg. In addition, data on secondary outcomes, including 60-day mortality, 90-day mortality and ICU mortality in patients with P/F ≤ 300 mmHg, were assessed. Other information is shown in Additional file 1: Table S1, including age, sex, ICU length of stay, days on MV, number of consecutive days of PP, number of cases of organ dysfunction, plateau pressure and V_t_. In addition, we noted the number of ARDS patients with direct lung injuries caused by pneumonia, aspiration, pulmonary contusion and other lung diseases, but not sepsis, shock, coma or postoperative causes.

**Table 1:** **Characteristics of the included patients**^**a**^

**Trial**	**Gattinoni *et al*., 2001**[19]	**Guerin *et al*., 2004**[14]	**Voggenreiter *et al*., 2005**[20]	**Curley *et al*., 2005**[18]	**Mancebo *et al*., 2006**[21]	**Chan *et al*., 2007**[22]	**Fernandez *et al*., 2008**[23]	**Taccone *et al*., 2009**[24]	**Guérin *et al*., 2013**[13]
Design	RCT	RCT	RCT	RCT	RCT	RCT	RCT	RCT	RCT
P/F for enrollment (mmHg)	300	300	300	300	200	300	300	200	150
Total number of included patients	304	791	40	101	136	22	40	342	466
PEEP level (cmH_2_O)	9	7	11	9	12	13	11	11	10
Duration of PP (h/day)	7.0	8.5	11	20	17	24	≥20	≥20	17
V_t_ (ml/kg)	10	8	6-8	7	8	7	7	7	6
28- to 30-day mortality in P/F ≤ 100 mmHg group (P (*n*/*N*), S (*n*/*N*))	NA^b^	NA	NA	NA	22/43, 21/29	NA	NA	28/74, 35/76	25/121, 41/121
28- to 30-day mortality in 100 ≤ P/F < 200 mmHg group (P (*n*/*N*), S (*n*/*N*))	NA	NA	NA	NA	11/33, 14/31	NA	NA	24/94, 22/98	13/116, 34/108
28- to 30-day mortality in P/F ≤ 300 mmHg group (P (*n*/*N*), S (*n*/*N*))	74/152, 70/152	134/413, 119/378	NA	4/51, 4/50	30/76, 32/60	7/11, 7/11	NA	52/168, 57/174	38/237, 75/229
60-day mortality in P/F ≤ 300 mmHg group (P (*n*/*N*), S (*n*/*N*))	95/152, 89/152	NA	NA	NA	22/76, 28/60	NA	8/21, 10/19	79/168, 91/174	NA
90-day mortality in P/F ≤ 300 mmHg group (P (*n*/*N*), S (*n*/*N*))	89/152, 84/152	179/413, 159/377	1/21, 3/19	NA	NA	NA	NA	NA	56/237, 94/229
ICU mortality in P/F ≤ 300 mmHg group (P (*n*/*N*), S (*n*/*N*))	77/152, 73/152	NA	NA	NA	33/76, 35/60	NA	NA	64/168, 73/174	NA

^a^ARDS, Acute respiratory distress syndrome; MV, Mechanical ventilation; *N*, Total number in group; *n*, Number of deaths; NA, Not available; P, Prone; P/F, Ratio of partial pressure of arterial oxygen to fraction of inspired of oxygen; PEEP, Positive end-expiratory pressure; S, Supine; V_t_, Tidal volume. ^b^Data not supplied in primary article.

Various P/F thresholds were used for patient enrollment, as Table 1 shows. In seven of the nine trials, investigators enrolled patents with P/F ≤ 300 mmHg [14,18-20,22,23]. In the trial by Guérin *et al*. [13], P/F was limited to ≤150 mmHg. Mancebo *et al*. [21] screened patents with P/F ≤ 200 mmHg, as did Taccone *et al*. [24]. Furthermore, the included trials applied different PEEP levels and PP durations. The PEEP levels in the included trials ranged from 7 to 13 cmH_2_O. The PEEP levels in three studies—by Gattinoni *et al*. [19], Guérin *et al*. [14] and Curley *et al*. [18]—were relatively low (<10 cmH_2_O) compared with those assessed in the others. Similarly, the PP duration varied from 7 to 24 h/day. In the studies by Gattinoni *et al*. [19], Guerin *et al*. [14] and Voggenreiter *et al*. [20], the patients were ventilated for markedly shorter PP durations (7 to 11 h/day) compared with patients in the other trials (17 to 24 h/day).

Additional file 2: Table S2 presents detailed information about the quality assessment of the included studies, including the Jadad score and the results of allocation concealment. All of the studies received scores of three points using the methods recommended by Jadad *et al*. Eight of the nine trials concealed allocation, and the other [22] did not. There were no obvious disagreements between the two reviewers (κ = 0.25) during the trial selection process or the methodological quality assessment.

### Quantitative data synthesis

#### PP decreased mortality in severe ARDS, but not in mild to moderate ARDS

**Figure 2:**
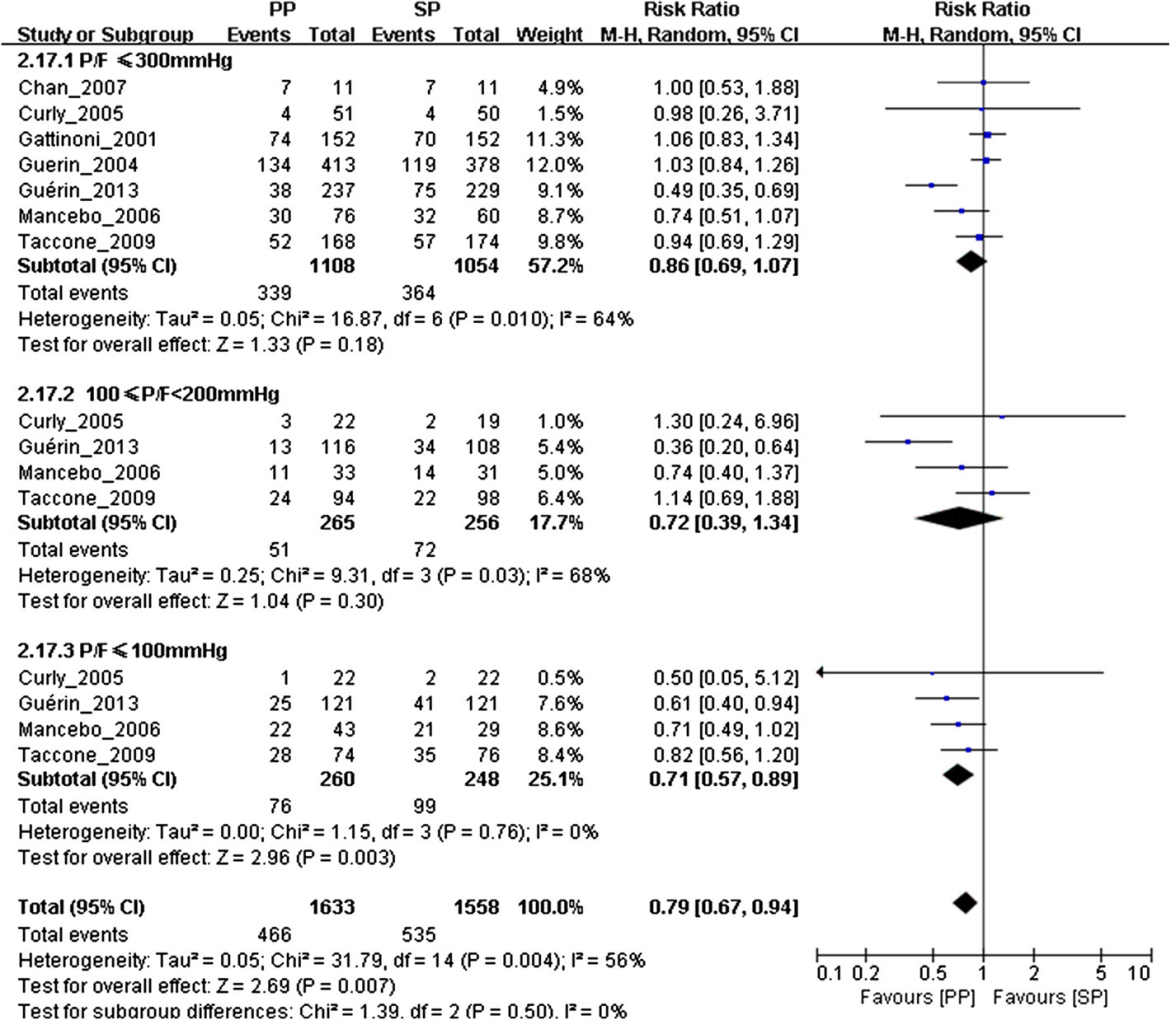
**Meta-analysis of the effect of prone positioning on 28- to 30-day mortality in acute respiratory distress syndrome patients related to the ratio of partial pressure of arterial oxygen/fraction of inspired oxygen.** The evidence gathered in our meta-analysis shows obvious heterogeneity, which was measured using the Mantel–Haenszel (M-H) χ^2^ test (*P* = 0.004) and the *I*^2^ heterogeneity test (*I*^2^ = 56%). A random-effects model was used. The *z*-test result for overall effects was not statistically significant (*P* = 0.007). In the ≤300 mmHg ratio of partial pressure of arterial oxygen/fraction of inspired oxygen (P/F) subgroup, the *z*-test for overall effects was not statistically significant (*P* = 0.18). In the subgroup of patients with P/F ratios between 100 and 200 mmHg, the *z*-test result for overall effects was not statistically significant (*P* = 0.30). In the subgroup of patients with P/F ≤ 100 mmHg, the *z*-test result for overall effects was statistically significant (*P* = 0.003). “Weight” is the contribution of each study to the overall risk ratio. CI, Confidence interval; I2, Percentage of total variation across studies from between-study heterogeneity rather than by chance; PP, Prone positioning; SP, Supine positioning.

As shown in Table 1, for ARDS patients with P/F ≤ 300 mmHg, 28- to 30-day mortality rates were reported in seven trials, 60-day and 90-day mortality rates were reported in four trials and ICU mortality was reported in three trials. The results of our meta-analysis show that, among ARDS patients with P/F ≤ 300 mmHg, there were no significant differences between the prone position and supine position groups with regard to 28- to 30-day mortality (*n* = 2,162, RR = 0.86, 95% CI = 0.69 to 1.07; *P* = 0.18;) (Figure 2), 60-day mortality (*n* = 822, RR = 0.92, 95% CI = 0.81 to 1.05; *P* = 0.21) (see Additional file 3: Figure S7), 90-day mortality (*n* = 1,600, RR = 0.85, 95% CI = 0.62 to 1.18; *P* = 0.33) (see Additional file 4: Figure S8) or ICU mortality (*n* = 785, RR = 0.92, 95% CI = 0.79 to 1.08; *P* = 0.31) (see Additional file 5: Figure S9). The funnel plot indicates the presence of publication bias following funnel plot analysis (see Additional file 6: Figure S1, Additional file 7: Figure S2 and Additional file 8: Figure S3), but we found no obvious publication bias in the meta-analysis of ICU mortality (see Additional file 9: Figure S4).

Moreover, subgroup meta-analyses were performed to determine the effect of PP on specific groups of patients. In four trials, the investigators reported 28- to 30-day mortality rates of patients with P/F ≤ 100 mmHg, and patients with P/F between 100 and 200 mmHg were included in the subgroup meta-analysis. The subgroup meta-analysis showed that PP decreased the 28- to 30-day mortality of patients with P/F ≤ 100 mmHg (*n* = 508, RR = 0.71, 95% CI = 0.57 to 0.89; *P* = 0.003) (Figure 2). There was no significant difference between the prone position and supine position groups regarding 28- to 30-day mortality of patients with P/F ratios between 100 and 200 mmHg (*n* = 521, RR = 0.72, 95% CI = 0.39 to 1.34; *P* = 0.30) (Figure 2). Because of the unavailability of data, it was impossible to perform analyses to assess the effects of PP on 60-day, 90-day and ICU mortality among ARDS patients with P/F ratios between 100 and 200 mmHg and among ARDS patients with P/F ≤ 100 mmHg.

#### PP reduced 60-day and 90-day mortality in ARDS patients ventilated with relatively high PEEP

**Figure 3:**
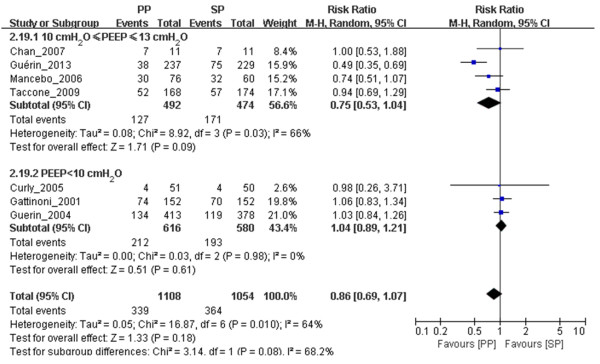
**Meta-analysis of the effect of prone positioning on 28- to 30-day mortality related to positive end-expiratory pressure in acute respiratory distress syndrome patients.** The evidence we gathered shows obvious heterogeneity, which we calculated using the Mantel–Haenszel (M-H) χ^2^ test (*P* = 0.01) and an *I*^2^ test (*I*^2^ = 64%). A random-effects model was used. The *z*-test result for overall effects was not statistically significant (*P* = 0.18). In the subgroup of patients with positive end-expiratory pressure (PEEP) < 10 cmH_2_O, the *z*-test result for overall effects was not statistically significant (*P* = 0.61). In the subgroup of patients with PEEP values between 10 and 13 cmH_2_O, the *z*-test result for overall effects was statistically significant (*P* = 0.09). “Weight” is the contribution of each study to the overall risk ratio. CI, Confidence interval; *I*^2^, percentage of total variation across studies from between-study heterogeneity rather than chance; PP, Prone positioning; SP, Supine positioning.

**Figure 4:**
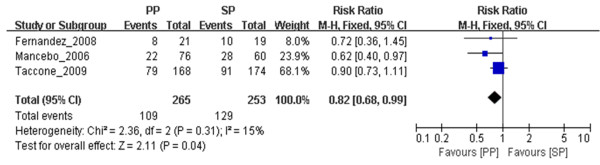
**Meta-analysis of the effect of prone positioning on 60-day mortality in acute respiratory distress syndrome patients with positive end-expiratory pressure ≥10 cmH**_**2**_**O.** No obvious heterogeneity was found using the Mantel–Haenszel (M-H) χ^2^ test (*P* = 0.31) and the *I*^2^ test (*I*^2^ = 15%). A fixed-effects model was used. The *z*-test result for overall effects was statistically significant (*P* = 0.04). “Weight” is the contribution of each study to the overall risk ratio. CI, Confidence interval; *I*^2^, Percentage of total variation across studies from between-study heterogeneity rather than chance; PP, Prone positioning; SP, Supine positioning.

**Figure 5:**
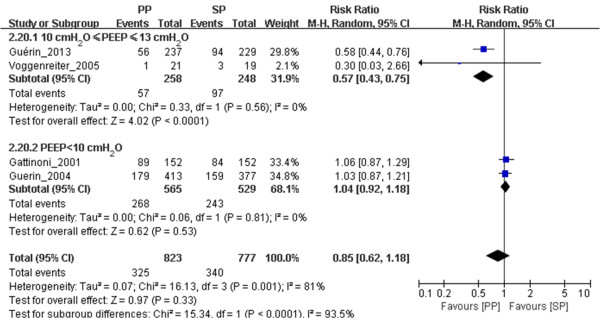
**Meta-analysis of the effect of prone positioning on 90-day mortality in acute respiratory distress syndrome patients related to positive end-expiratory pressure.** The evidence we gathered shows obvious heterogeneity based on the results of the Mantel–Haenszel (M-H) χ^2^ test (*P* = 0.001) and the *I*^2^ test (*I*^2^ = 81%). A random-effects model was used. The *z*-test result for overall effects was statistically significant (*P* = 0.33) in the subgroup of patients with positive end-expiratory pressure (PEEP) levels ≥10 cmH_2_O. In the PEEP <10 cmH_2_O subgroup, the *z*-test result for overall effects was not statistically significant (*P* = 0.53). In the subgroup of patients with PEEP levels between 10 and 13 cmH_2_O, the *z*-test result for overall effects was statistically significant (*P* < 0.0001). “Weight” is the contribution of each study to the overall risk ratio. CI, Confidence interval; *I*^2^, Percentage of total variation across studies from between-study heterogeneity rather than chance; PP, Prone positioning; SP, Supine positioning.

We included in our meta-analysis seven trials in which 28- to 30-day mortality was reported, three trials in which 60-day mortality was reported and four trials in which 90-day mortality was reported (Table 1), with PEEP thresholds as high as 10 cmH_2_O. Although we found no significant difference in 28- to 30-day mortality (*n* = 966, RR = 0.75, 95% CI = 0.53 to 1.04; *P* = 0.09) (Figure 3), we did find significant differences in both 60-day mortality (*n* = 518, RR = 0.82, 95% CI = 0.68 to 0.99; *P* = 0.04) (Figure 4) and 90-day mortality (*n* = 506, RR = 0.57, 95% CI = 0.43 to 0.75; *P* < 0.0001) (Figure 5) between the prone position and supine position groups with 10 cmH_2_O ≤ PEEP ≤ 13 cmH_2_O. We found no significant differences in either 28- to 30-day mortality (*n* = 1,196, RR = 1.04, 95% CI = 0.89 to 1.21; *P* = 0.61) (Figure 3) or 90-day mortality (*n* = 1,094, RR = 1.04, 95% CI = 0.92 to 1.18; *P* = 0.56) (Figure 5) between the prone position and supine position groups with PEEP < 10 cmH_2_O. No obvious publication bias was found (see Additional file 10: Figure S5), except for the subgroup analysis of 90-day mortality related to PEEP (see Additional file 11: Figure S6). Because of insufficient data, we did not analyze the effects of PP on ICU mortality of patients with 10 cmH_2_O ≤ PEEP ≤ 13 cmH_*2*_O or PEEP < 10 cmH_2_O.

#### PP reduced 28-day to 30-day mortality when PP duration was longer than 12 hours/day

**Figure 6:**
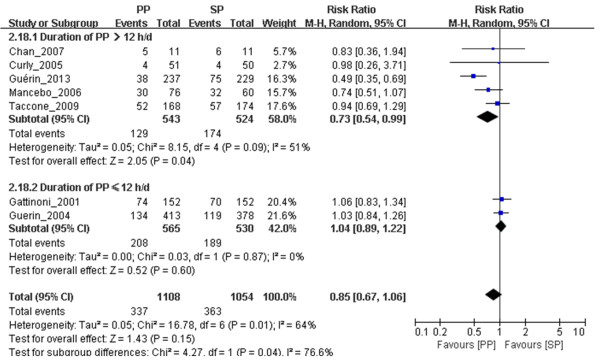
**Meta-analysis of the effect of prone positioning on 28- to 30-day mortality in acute respiratory distress syndrome patients related to the duration of prone positioning.** The evidence we gathered shows obvious heterogeneity based on the results of the Mantel–Haenszel (M-H) χ^2^ test (*P* = 0.01) and the *I*^2^ test (*I*^2^ = 64%). A random-effects model was used. The *z*-test result for overall effects was not statistically significant (*P* = 0.15). In the subgroup of patients with prone positioning (PP) duration ≥12 h/day, the *z*-test result for overall effects was statistically significant (*P* = 0.04). In the subgroup of patients with PP duration <12 h/day, the *z*-test result for overall effects was statistically significant (*P* = 0.60). “Weight” is the contribution of each study to the overall risk ratio. CI, Confidence interval; *I*^2^, Percentage of total variation across studies from between-study heterogeneity rather than chance; SP, Supine positioning.

We included in the meta-analysis seven trials in which the investigators reported 28- to 30-day mortality, which we stratified according to PP duration, with a threshold of 12 h/day. The funnel plots (see Additional file 6: Figure S1) indicate a possible publication bias. No significant differences were found in 28- to 30-day mortality between the PP and SP groups when the PP duration was ≤12 h/day (*n* = 1,095, RR = 1.04, 95% CI = 0.89 to 1.22; *P* = 0.60) (Figure 6). Among patients with PP durations >12 h/day, however, we found a significant decrease in 28- to 30-day mortality in the PP group (*n* = 1,067, RR = 0.73, 95% CI = 0.54 to 0.99; *P* = 0.04) (Figure 6) compared with the SP group. The effects of PP on 90-day and ICU mortality were not analyzed, owing to insufficient data.

## Discussion

The first finding of our meta-analysis is that PP decreases 28- to 30-day mortality in severe ARDS patients (defined as a baseline P/F ≤ 100 mmHg), but not in moderate ARDS patients. These results confirm what was suggested by a previous meta-analysis [12], which is that the main benefit of PP is observed in patients with P/F ≤ 100 mmHg. This phenomenon has been suggested to be based primarily on the association between PP and a decreased risk of lung injury due to stress and strain forces [12,25]. Patients with severe ARDS are at the greatest risk of lung injury from shear and strain forces because of a low ratio of well-aerated lung tissues to poorly aerated or nonaerated lung tissues [12,26]. When a patient is placed in the prone position, the lung has greater homogeneity and stress and strain forces are decreased.

The second finding of our meta-analysis is that PP reduced both 60- and 90-day mortality in the groups of ARDS patients who were ventilated with relatively high PEEP levels (10 cmH_2_O ≤ PEEP ≤ 13 cmH_2_O). There are at least three possible explanations for this finding: (1) high PEEP levels might be merely a marker of severity, similar to the P/F ratio; (2) high PEEP levels might increase the risk of ventilator-associated lung injury in nonrecruitment conditions (increased hyperinflation); and (3) PP and PEEP might exert additive or synergetic protective effects. Cornejo *et al*. [27] reported that PP enhanced the effects of high PEEP levels in terms of lung recruitment and reductions in cyclic recruitment/derecruitment, whereas it prevented the negative impact of PEEP on tidal hyperinflation. Because ARDS is a heterogeneous syndrome, these possibilities are not mutually exclusive.

The third finding of our meta-analysis is that PP reduced 28- to 30-day mortality in ARDS patients with relatively long PP durations (defined as PP duration >12 h/day). Researchers in several previous studies have suggested that PP duration should be considered when assessing the effects of PP, because alveolar recruitment in the prone position is a time-dependent event [28]. However, the results of other previous clinical investigations have failed to confirm this finding [11]. The results of our present meta-analysis show that the mortality rate in the prone position group (129 (23.76%) of 543 patients) was significantly lower than that in the supine position group (208 (33.81%) of 565 patients), indicating that PP duration also played an important role in the survival advantage associated with PP. However, it is unclear whether this finding is due to a dose response to PP or whether a threshold daily PP duration is required to obtain a benefit. Moreover, we have no evidence indicating which patients benefited the most from long-term PP.

Our meta-analysis has some limitations. It is likely that we did not include all of the evidence, because we limited our analysis to articles in the English-language literature. Another limitation is associated with the data that we obtained from the nine included trials. Some of the trials reported the duration of PP only with medians and interquartile ranges. We estimated the means and variances based on the medians, ranges and sizes of the trials using the formulas recommended by Hozo *et al*. [29]. In addition, we used the mean overall duration of daily PP in each included trial in this trial-level analysis. This might have resulted in ecological bias [30]. The small sample size may also have been a limitation, especially in the subgroup analyses with few included patients. Moreover, the variability in the selection criteria for RCTs and sample size, the incomplete reporting of intervention intensity, the use of low-V_t_ ventilation and the absence of volume–outcome relationships in patients with ARDS may also be limitations.

## Conclusions

Similar to a previous meta-analysis [12], our present study-level meta-analysis shows that PP significantly reduced mortality in severe ARDS patients. However, we found no demonstrated benefit of PP in patients with mild to moderate ARDS. The new contribution of our meta-analysis is the finding that PP decreased mortality in ARDS patients who received relatively high PEEP levels. Furthermore, we found that long-term PP reduced mortality in ARDS patients, indicating that PP duration also plays an important role in the survival advantage of PP. It is unclear if this importance is a result of a dose response to PP or whether there existed a threshold daily PP duration required to obtain a benefit. The data we gathered suggest that PP <12 h/day is less likely to be beneficial to ARDS patients.

## Key messages

• Patients with severe ARDS (defined as P/F ratio ≤100 mmHg) clearly benefit from PP.

• There is no demonstrated benefit of PP in patients with mild to moderate ARDS.

• Patients ventilated with a higher PEEP level (defined as PEEP ≥10 cmH_2_O) also benefit from PP. Because the results of this study do not allow a definitive explanation for these findings, no firm recommendations can be made regarding the use of PP based on PEEP level.

• In this study, we show that the PP duration matters. It is unclear whether this importance resulted from a dose response to PP or whether there existed a threshold daily PP duration that was required to obtain a benefit. The data suggest, however, that PP for <12 h/day is less likely to be beneficial to patients.

## Abbreviations

ARDS: Acute respiratory distress syndrome; CI: Confidence interval; M-H: Mantel–Haenszel; MV: Mechanical ventilation; P/F: Ratio of partial pressure of arterial oxygen to inspired fraction of oxygen; PEEP: Positive end-expiratory pressure; PP: Prone positioning; RCT: Randomized controlled trial; RR: Risk ratio; SP: Supine positioning; V_t_: Tidal volume.

## Competing interests

The authors declare that they have no competing interests.

## Authors’ contributions

SLH conducted the literature searches, study selection, data extraction and study quality assessment and also prepared the initial drafts of the manuscript and revised it according to advice from the other authors. HLH, ARL and CP reviewed abstracts, selected studies meeting the inclusion criteria, extracted data and assessed study quality. SLH, SQL and LL input data and performed the statistical analyses. YZH and FMG helped to synthesize data and provided methodological guidance on the use of RevMan 5.2.3 software. YY and HBQ were responsible for the design of the work and revised the manuscript for important intellectual content. All authors reviewed and approved the final manuscript.

## Supplementary Material

Additional file 1: Table S1 Supplementary data of the included patients. Click here for file

Additional file 2: Table S2 Methodology quality assessment. Click here for file

Additional file 3: Figure S7 Meta-analysis of the effect of PP on 60-day mortality in ARDS patients with P/F ≤300 mmHg. Click here for file

Additional file 4: Figure S8 Meta-analysis of the effect of PP on 90-day mortality in ARDS patients with P/F ≤300 mmHg. Click here for file

Additional file 5: Figure S9 Meta-analysis of the effect of PP on ICU mortality in ARDS patients with P/F ≤300 mm Hg. Click here for file

Additional file 6: Figure S1 Funnel plot for meta-analysis of the effect of PP on 28- to 30-day mortality in ARDS patients related to P/F. Click here for file

Additional file 7: Figure S2 Funnel plot for meta-analysis of the effect of PP on 60-day mortality in ARDS patients with P/F ≤300 mmHg. Click here for file

Additional file 8: Figure S3 Funnel plot for meta-analysis of the effect of PP on 90-day mortality in ARDS patients with P/F ≤300 mmHg). Click here for file

Additional file 9: Figure S4 Funnel plot for meta-analysis of the effect of PP on ICU mortality in ARDS patients with P/F ≤300 mmHg. Click here for file

Additional file 10: Figure S5 Funnel plot for subgroup meta-analysis of the effect of PP on 60-day mortality in ARDS patients with PEEP ≥10 cmH_2_O. Click here for file

Additional file 11: Figure S6 Funnel plot for subgroup meta-analysis of the effect of PP on 90-day mortality in ARDS patients related to PEEP. Click here for file
