# Supplementary material for: The effect of prone positioning on mortality in patients with acute respiratory distress syndrome: a meta-analysis of randomized controlled trials
Source: Crit Care. 2014 May 28;18(3):R109. doi: 10.1186/cc13896 (PMC4075407; doi:10.1186/cc13896)
Supplement: Additional file 1: Table S1 — Supplementary data of the included patients. [file cc13896-S1.pdf]

**Table S1** Characteristics of the included patients

| Trial | Age (yr) ( P, S) | Female (%)( P, S) | ICU LOS[#] (days) ( P, S) | Days of MV ( P, S) | Direct pulmonary injury (%)( P, S) | Pplat cm $H_2O$ | Paralytics | HFO | No. of days[¤] | No. of organ dysfunctions ( P, S) |
|---|---|---|---|---|---|---|---|---|---|---|
| **Gattinoni 2001(9)** | 59±17, 57±16 | 34.2, 25 | NA[※] | NA | >80.4, >71.4 | NA | YES | NO | 10 | 1.4±1.0[※], 1.3±1.0 |
| **Guerin 2004(10)** | 62.0±15.7, 62.5±14.7 | 26.4, 23.5 | 26.6±29.6, 24.5±21.9 | 13.7±7.8, 14.1±8.6 | >61.7, >60.3 | NA | YES | NO | 4 | 2.2±1.0[※], 2.3±1.0 |
| **Voggenreiter 2005(18)** | 40±14, 43±10 | 14, 21 | NA | 30±17, 33±23 | NA | NA | YES | NO | 10 | NA |
| **Curley 2005(17)** | 2.0, 2.1 | 53, 41 | NA | NA | 82, 86 | NA | NA | YES | 7 | 2, 2 |
| **Mancebo 2006(22)** | 54±17, 54±16 | 30, 42 | 20.5±18.2, 19.1± 23.1 | NA | >70, >52 | 32 | YES | NO | 10 | 1.1±1.2, 1.1±1.2 |
| **Chan 2007(19)** | 54.7 ±21.8,69.9 ±15.5 | 18.2, 18.2 | NA | NA | NA | NA | NA | NO | ⩾3 | NA |
| **Fernandez 2008(20)** | 53.9±17.9, 55.3±14.6 | 43, 31 | 14.7±9.7, 17.5±16.1 | 11.9±9, 15.7±16.9 | >62, >68 | 29.2 | NA | NO | NA | NA |
| **Taccone 2009(25)** | 60±16 | 28.7 | 17.5, 16 | 16, 14 | >65.5, >65.5 | ⩾20 | YES | NO | 28 | NA |
| **Guérin 2013(14)** | 58±16, 60±16 | 30, 33.6 | 22.5, 22 | NA | >62.4 | 23.5 | YES | NO | 28 | NA |

※Data were not supplied in the primary article; P, prone; S, supine; ICU, intensive care unit; n, number of deaths; N, number in group; ＃ICU long of stay; ¤ Number of days that prone-position occurred; MV, mechanical ventilation; Pplat, plateau pressure
