# Supplementary material for: The effect of prone positioning on mortality in patients with acute respiratory distress syndrome: a meta-analysis of randomized controlled trials
Source: Crit Care. 2014 May 28;18(3):R109. doi: 10.1186/cc13896 (PMC4075407; doi:10.1186/cc13896)
Supplement: Additional file 2: Table S2 — Methodology quality assessment. [file cc13896-S2.pdf]

Table S2 Methodology quality assessment

Trial	Randomization	Blinding	Handling of withdrawals and dropouts	Jadad score	Allocation concealment	Crossover
Gattinoni2001(9)	2	0	1	3	adequate	YES
Guerin2004(10)	2	0	1	3	adequate	YES
Voggenreiter2005(18)	2	0	1	3	adequate	NO
Curley2005(17)	2	0	1	3	adequate	YES
Mancebo2006(22)	2	0	1	3	adequate	YES
Chan2007(19)	2	0	1	3	not used	NO
Fernandez2008(20)	2	0	1	3	adequate	YES
Taccone2009(25)	2	0	1	3	adequate	YES
Guérin2013(14)	2	0	1	3	adequate	YES
