# Supplementary material for: The effect of prone positioning on mortality in patients with acute respiratory distress syndrome: a meta-analysis of randomized controlled trials
Source: Crit Care. 2014 May 28;18(3):R109. doi: 10.1186/cc13896 (PMC4075407; doi:10.1186/cc13896)
Supplement: Additional file 3: Figure S7 — Meta-analysis of the effect of PP on 60-day mortality in ARDS patients with P/F ≤300 mmHg. [file cc13896-S3.pdf]

Study or Subgroup	PP Events	PP Total	SP Events	SP Total	Weight	Risk Ratio M-H, Fixed, 95% CI
Fernandez_2008	8	21	10	19	4.8%	0.72 [0.36, 1.45]
Gattinoni_2001	95	152	89	152	40.4%	1.07 [0.89, 1.28]
Mancebo_2006	22	76	28	60	14.2%	0.62 [0.40, 0.97]
Taccone_2009	79	168	91	174	40.6%	0.90 [0.73, 1.11]
**Total (95% CI)**		**417**		**405**	**100.0%**	**0.92 [0.81, 1.05]**
Total events	204		218			
Heterogeneity: Chi² = 6.11, df = 3 (P = 0.11); I² = 51%						
Test for overall effect: Z = 1.26 (P = 0.21)						

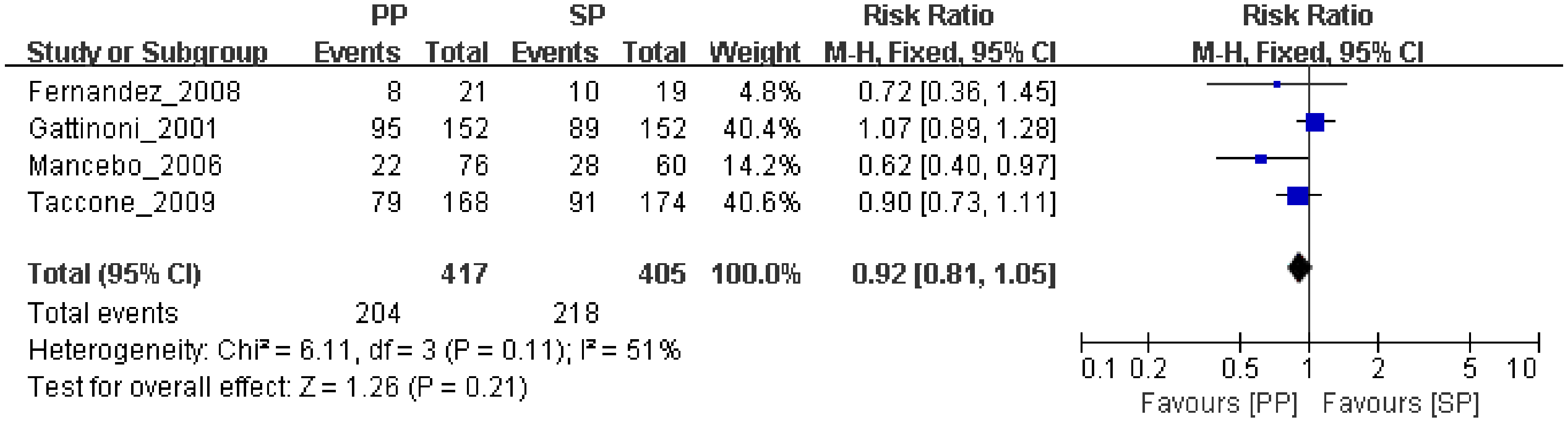

Figure S7 Meta-analysis of the effect of PP on 60-day mortality in ARDS patients with P/F ≤ 300mmHg. The I^2 test shows that I^2 (I^2 = 51%) is around 50%. However, the M-H chi-square test (p = 0.11) showed no obvious heterogeneity. Hence, fixed-effects model was performed. The z test for overall effects was not statistically significant (p = 0.21). Weight is the contribution of each study to the overall RR. PP, prone positioning; SP, supine prone; ARDS, acute respiratory distress syndrome; P/F, partial pressure of arterial oxygen/inspired fraction of oxygen; M-H, Mantel-Haenzel; I^2, percentage of total variation across studies from between-study heterogeneity rather than chance; RR, risk ratio; CI, confidence interval .
