# Supplementary material for: The effect of prone positioning on mortality in patients with acute respiratory distress syndrome: a meta-analysis of randomized controlled trials
Source: Crit Care. 2014 May 28;18(3):R109. doi: 10.1186/cc13896 (PMC4075407; doi:10.1186/cc13896)
Supplement: Additional file 4: Figure S8 — Meta-analysis of the effect of PP on 90-day mortality in ARDS patients with P/F ≤300 mmHg. [file cc13896-S4.pdf]

	PP		SP			Risk Ratio
Study or Subgroup	Events	Total	Events	Total	Weight	M-H, Random, 95% CI
Gattinoni_2001	89	152	84	152	33.4%	1.06 [0.87, 1.29]
Guerin_2004	179	413	159	377	34.8%	1.03 [0.87, 1.21]
Guérin_2013	56	237	94	229	29.8%	0.58 [0.44, 0.76]
Voggenreiter_2005	1	21	3	19	2.1%	0.30 [0.03, 2.66]
**Total (95% CI)**		**823**		**777**	**100.0%**	**0.85 [0.62, 1.18]**
Total events	325		340			

Heterogeneity: Tau² = 0.07; Chi² = 16.13, df = 3 (P = 0.001); I² = 81%
Test for overall effect: Z = 0.97 (P = 0.33)

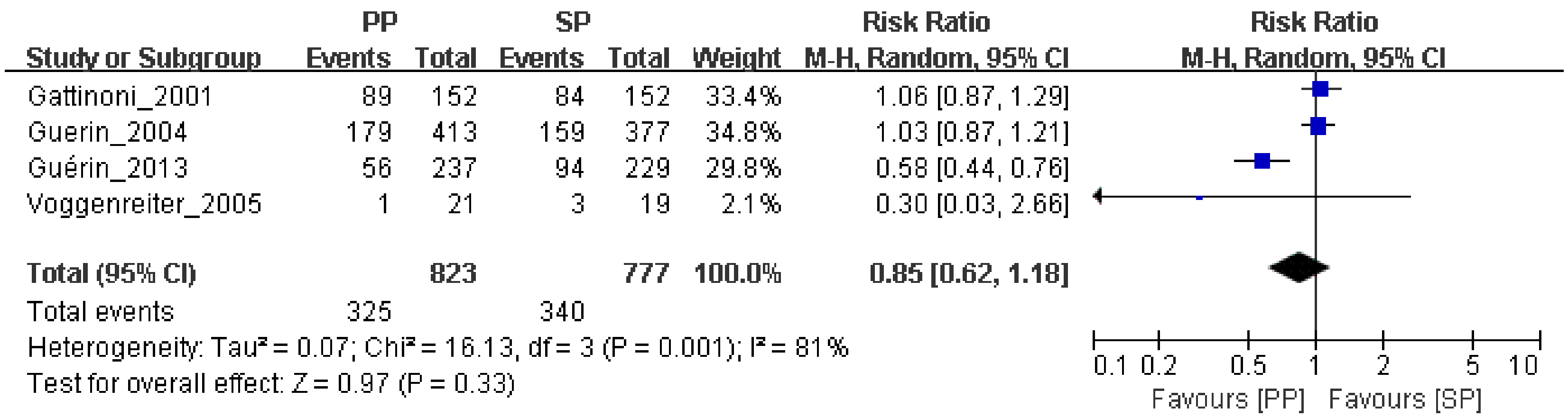

Figure S8 Meta-analysis of the effect of PP on 90-day mortality in ARDS patients with P/F ≤ 300 mm Hg. Evidence showed obvious heterogeneity using the M-H chi-square test (p = 0.001) and the I^2 test (I^2 = 81%). Random-effects model was performed. The z test for overall effects was not statistically significant (p = 0.33). Weight is the contribution of each study to the overall RR. PP, prone positioning; SP, supine prone; ARDS, acute respiratory distress syndrome; P/F, partial pressure of arterial oxygen/inspired fraction of oxygen; M-H, Mantel-Haenzel; I^2, percentage of total variation across studies from between-study heterogeneity rather than chance; RR, risk ratio; CI, confidence interval.
