# Supplementary material for: The effect of prone positioning on mortality in patients with acute respiratory distress syndrome: a meta-analysis of randomized controlled trials
Source: Crit Care. 2014 May 28;18(3):R109. doi: 10.1186/cc13896 (PMC4075407; doi:10.1186/cc13896)
Supplement: Additional file 5: Figure S9 — Meta-analysis of the effect of PP on ICU mortality in ARDS patients with P/F ≤300 mm Hg. [file cc13896-S5.pdf]

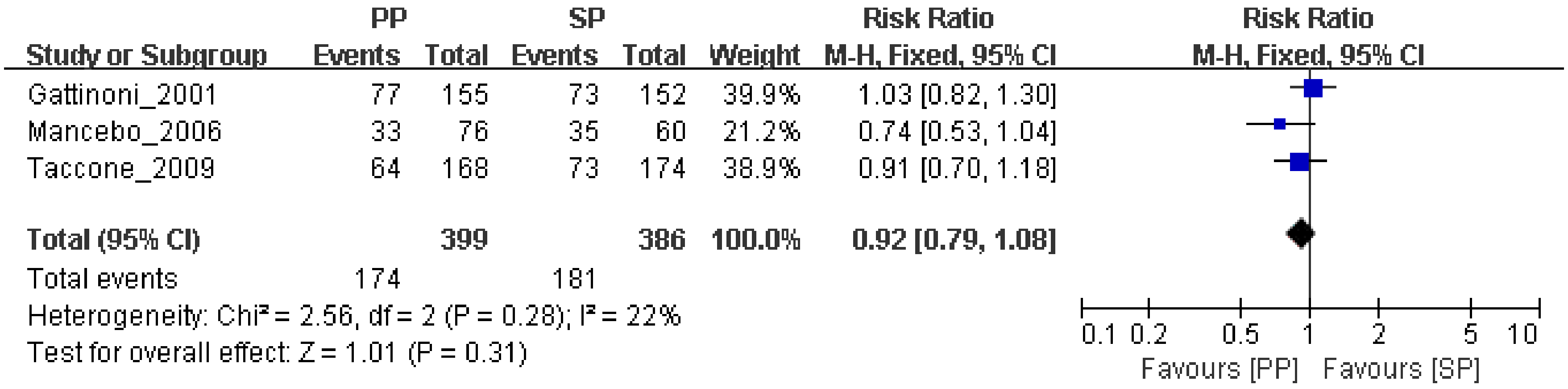

Figure S9 Meta-analysis of the effect of PP on ICU mortality in ARDS patients with P/F ≤ 300 mm Hg. No obvious heterogeneity was found using the M-H chi-square test (p = 0.28) and the I^2 test (I^2 = 22%). Fixed-effects model was performed. The z test for overall effects was not statistically significant (p = 0.31). Weight is the contribution of each study to the overall RR. PP, prone positioning; SP, supine prone; ARDS, acute respiratory distress syndrome; P/F, partial pressure of arterial oxygen/inspired fraction of oxygen; M-H, Mantel-Haenzel; I^2, percentage of total variation across studies from between-study heterogeneity rather than chance; RR, risk ratio; CI, confidence interval .
