# Supplementary material for: The effect of prone positioning on mortality in patients with acute respiratory distress syndrome: a meta-analysis of randomized controlled trials
Source: Crit Care. 2014 May 28;18(3):R109. doi: 10.1186/cc13896 (PMC4075407; doi:10.1186/cc13896)
Supplement: Additional file 6: Figure S1 — Funnel plot for meta-analysis of the effect of PP on 28- to 30-day mortality in ARDS patients related to P/F. [file cc13896-S6.pdf]

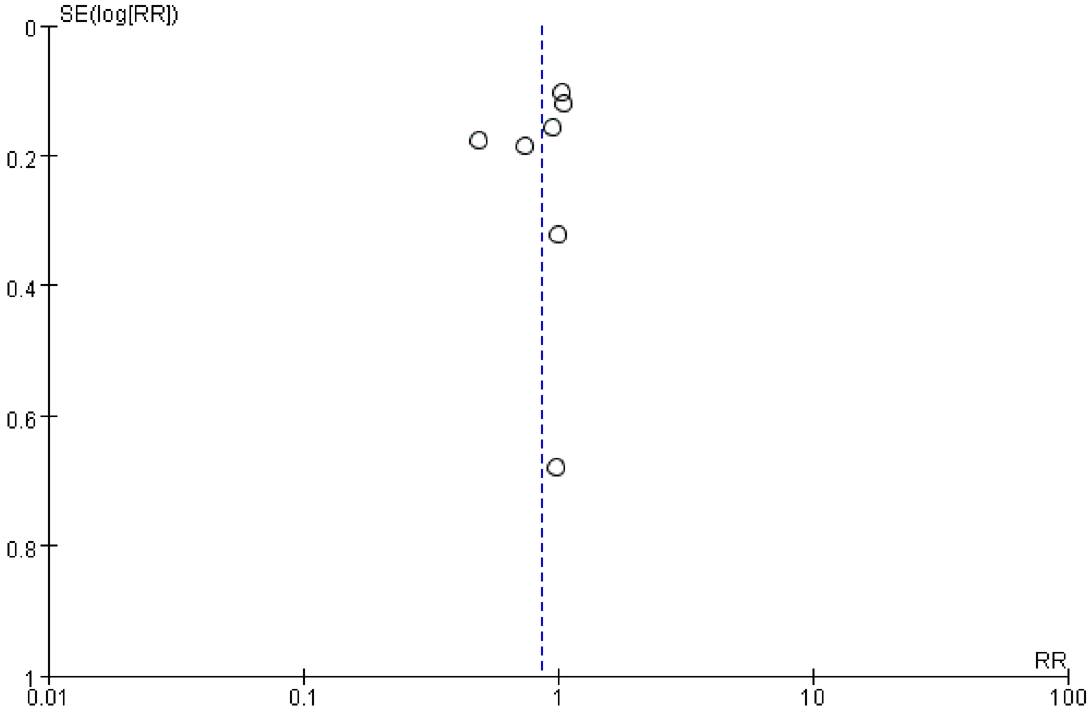

Figure S1 Funnel plot for meta-analysis of the effect of PP on 28- to 30-day mortality in ARDS patients related to P/F.

The funnel plot showed the plots were asymmetrical distributed, indicating a possible publication bias. Each point represents one trial.

RR, risk ratio; PP, prone positioning; ARDS, acute respiratory distress syndrome; P/F, partial pressure of arterial oxygen/ inspired fraction of oxygen
