# Supplementary material for: The effect of prone positioning on mortality in patients with acute respiratory distress syndrome: a meta-analysis of randomized controlled trials
Source: Crit Care. 2014 May 28;18(3):R109. doi: 10.1186/cc13896 (PMC4075407; doi:10.1186/cc13896)
Supplement: Additional file 10: Figure S5 — Funnel plot for subgroup meta-analysis of the effect of PP on 60-day mortality in ARDS patients with PEEP ≥10 cmH2O. [file cc13896-S10.pdf]

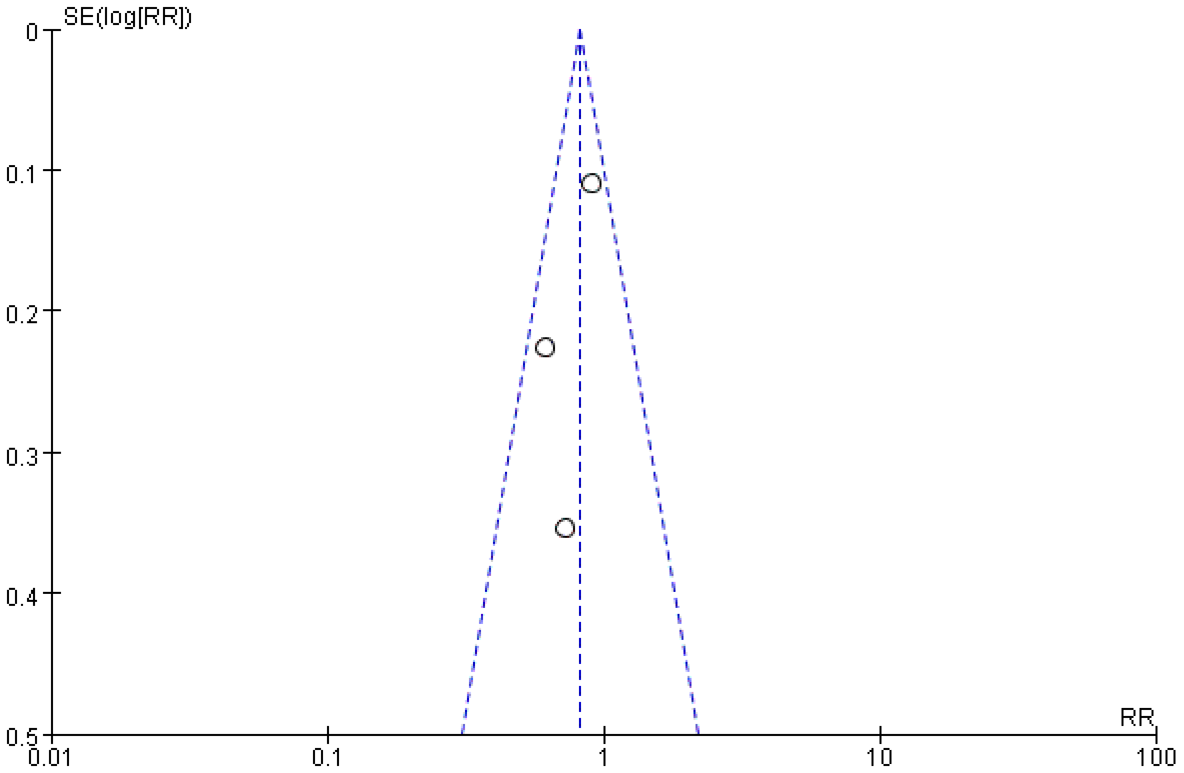

Figure S5 Funnel plot for subgroup meta-analysis of the effect of PP on 60 days mortality in ARDS patients with PEEP ≥ 10 cm H_2O.

The funnel plot showed the plots were symmetrical distributed, indicating no obvious publication bias. Each point represents one trial.

RR, risk ratio; PP, prone positioning; ARDS, acute respiratory distress syndrome; PEEP, positive end-expiratory pressure
